# Supplementary material for: Distortion correction of diffusion weighted MRI without reverse phase-encoding scans or field-maps
Source: PLoS One. 2020 Jul 31;15(7):e0236418. doi: 10.1371/journal.pone.0236418 (PMC7394453; doi:10.1371/journal.pone.0236418)
Supplement: Attachment — Submitted filename: R1_Response2Reviewers.docx [file pone.0236418.s001.docx]

­­Dear Dr. Yap,

Please find attached a revision in response to the concerns raised by the reviewers. Major changes include additional text addressing reviewer concerns and appropriate additional discussion, as well as major changes to figures in order to increase clarity. Finally, additional analysis was performed to strengthen the claim of generalizability. The text that directly responds to the reviewers’ concerns is highlighted and annotated below, and is marked in the revised manuscript.

Kind regards,

Kurt Schilling

R1.C1: The paper describes a learning-based distortion correction framework, where the structural T1 and distorted single-blip B0 images are used to synthesize undistorted B0 images. Later, the synthesized and distorted B0 images are fed into TOPUP for further correction. Lastly, FSL's eddy tool is used to do complete pre-processing of the DWIs.

Primary flaws of the paper are 1) presentation of the method, 2) novelty & technical soundness.

We appreciate the feedback and have made major revisions to textual descriptions of the methods figures. We have further highlighted the novelty throughout the text. Without additional acquisitions (reverse PE or field map) no method currently exists to correct for susceptibility distortions, except for image registration which comes with its own difficulties (associated with constraints in PE direction, signal pile-up, etc.). We propose a solution to this problem.

R1.C2: In Section 1, the authors highlighted the limitations of their previous work (Synb0-DisCo) but didn't show how these limitations are addressed by the proposed approach.

We now explicitly state the limitations and how they are addressed (Introduction).

While Synb0-DisCo is a promising first approach for a deep learning solution to the DW-MRI distortion correction problem, it has several limitations. First, Synb0-DisCo does not intrinsically compensate for absolute intensities of the target minimally weighed scans, and therefore, secondary adjustment of the intensity spaces is needed. Second, patient specific contrasts seen in the acquired distorted DW-MRI cannot be learned as the network only had relatively homogeneous T1w MRI information available. Third, Synb0-DisCo is susceptible to 3D inconsistencies as the model did not have access to full imaging context.

Herein, we propose a second generation of our deep learning approach, termed Synb0, for DW-MRI distortion correction to address these limitations. Briefly, we (1) generalize the learning approach to use both T1w and distorted DW-MRI images in order to synthesize a b0 with both appropriate geometry and contrast, (2) redesign the network to use full 3-D information, and (3) train across a much larger collection of patients / studies / scanners in order to facilitate generalization across different datasets with varying acquisitions and cohorts.

R1.C3: The title of the paper is misleading ("Registration-free"..), as the method relies on TOPUP and it is not truly registration-free. Also, I really don't get the idea of using TOPUP on top of B0 synthesis. If the synthetic undistorted B0 image is good enough then why run TOPUP?

We agree and have changed the title of the manuscript. While we do not use “registration” in the traditional sense, we do utilize the advanced features of TOPUP (estimating displacement by minimizing a cost function associated with image intensities) that do have a registration step to estimate subject motion.

Additionally, we now clearly describe why we have to use TOPUP (beginning of Methods section). The rationale here is that while we create an undistorted image, we actually use TOPUP to perform the correction:

Put another way, when application of topup and its advanced distortion correction features and assumptions would traditionally not be possible (due to the absence of reverse PE scans or field maps), we are synthesizing a geometrically *undistorted* image in order to provide topup the information necessary to perfectly correct the *distorted* diffusion data.

R1.C4: It is difficult to understand the loss functions in textual form, so it would be better if the authors formulate all the loss functions mathematically.

We have now written out the loss function (the averaging of two MSE losses: $\mathcal{l}$_1_ + $\mathcal{l}$_2_ / 2) in the Section 2.3 Network/Training/Loss. In addition, per R1.C5 and R2.C6, we have significantly modified Figure 4 for both better visualization and to remove redundant (and confusing) text from the diagram. We believe the figure in combination with the text significant clarifies the loss function(s).

R1.C5: All the figures are of poor quality. The text in the boxes of Fig. 4 is illegible. There is no network architecture shown. The surface overlays in Fig. 6 & 8 are also not clear.

We have made significant changes to all figures. For Figure 4 we have removed sub-scripts that were redundant and also modified the diagram (R1.C4). Additionally, all surface overlays have been removed entirely because they were not clear. Instead, we show both the distorted and undistorted (after application of our pipeline) b0 images that qualitatively indicate the efficacy of the pipeline (which is quantified in further figures).

R2.C1: The authors propose a novel method to predict an undistorted b0 from a distorted b0 and a structural image using deep learning (U-nets). This undistorted b0 is then used as a reference in FSL's topup to estimate the susceptibility field (i.e., fieldmap), which allows legacy data acquired without an alternative way to estimate the fieldmap to still be analysed. It's a well-presented and very promising approach to this problem.

We thank the reviewer for their encouraging review and constructive feedback.

R2.C2: My only issue is whether the algorithm will be useful in legacy datasets as this does require the U-net to generalise to other datasets. The authors claim in the abstract: "we show generalizability of the proposed approach to datasets that were not in the original training / validation / testing datasets". However, in the paper they only show 3 selected examples from 3 datasets, where the algorithm does indeed do a decent job. A more quantitative comparison of this claim could be easily produced from for example the distribution of the MI with the T1 (similar to what was used in the withheld dataset in the left panel in Figure 7). This would allow the authors to show whether the algorithm reliably generalizes to other dataset, which would be required before applying it to legacy datasets.

We agree that without generalizability this algorithm and pipeline is not useful to the scientific community at large. For this reason, we have downloaded 11 open-source diffusion datasets (with widely varying resolutions, T1 contrast, diffusion contrast, PE directions, and even including pediatric data) and show efficacious synthesis and distortion-correction in all datasets. We further quantify the MI with T1 and show that in all 11 specific examples that the MI increases and state this in the text. The pre- and post-correction images are shown in Figure 8 and we describe the datasets in the Methods section.

Minor issues:

R2.C3: Please give the units of the reconstructed voxel size in the BLSA dataset (page 3).

Units added to voxel size.

R2.C4: When mentioning the coregestration from b0 to T1 using epi_reg, it would be good to mention that this is a rigid-body transformation (6 degrees of freedom).

We have noted that this is a rigid-body 6 degrees of freedom transformation.

R2.C5: Was the T1 image registered to the standard after intensity normalization (implied by the text) or before intensity normalization (implied by Figure 3)?

Intensity normalization is done before registration. We now show this in blocks in the figure as well as describe normalization first in the text.

R2.C6: There are many arrows missing and misplace in Figure 4. The ones I spotted are:
       - There should be an arrow from b0_d_MNI_1 to the upper U-net (top part).
       - There should be an arrow from b0_u_MNI to the top MSE (top part).
       - There should be an arrow from b0_m_MNI_2 to middle MSE (top part).
       - Why is there an arrow going upwards from T1_norm_MNI (top part)?
       - In the lower part the arrow between b0_d_MNI_1 and the U-net is shifted upwards.

Thank you for taking the time and due diligence to assess this figure critically. We have completely revised this figure (removing subscripts, changing diagram order, etc.) which we believe improves clarity. In addition, we now believe that all missing and misplaced arrows have been corrected.

R2.C7: In the caption of Figure 6 there is no mention of which outline in the right panels is the distorted b0 and which is the undistored b0. This information is in the caption of Figure 8, but there it is the wrong way around (red is hopefully the outline of the undistorted b0). Also, in Figure 6, there are two typos, namely "With-held" should be "Withheld" and "let" should be "left".

We have removed outlines/overlays per R1.C5 and instead show corresponding slices adjacent to the T1 images. Now the images are clearly marked with a “D” or “U” for distorted/undistorted in both Figure 6 and 8. We have additionally fixed the typos.

R2.C8: What is the legacy synthetic distortion correction (S.D.) approach mentioned in the caption of Figure 7. It might be good to mention this in the results section when discussion Figure 7.

We now describe this in the results section when discussing Figure 7. We state (and cite) the legacy distortion correction and note:

We point out that the legacy distortion correction^33^ (S.D.) appears to show improvements mainly for the BLSA datasets (which it was trained on) and did not well generalize to additional contrasts and geometries – limitations which the currently proposed approach specifically intend to address.

R2.C9: When discussing the timings in the discussion, it would be good to mention that the full pipeline of Synb0 still involves running topup, so the total runtime of Synb0 is much more than 2 minutes.

We now state this in the discussion.

The improvement is consistent across multiple datasets. Moreover, Synb0 runs in ~2 minutes per scan (specifically, inference, or generation of synthetic images, is ~2 minutes), versus ~10-15 minutes for image-based registration. It is important to point out that the full proposed pipeline still involves running topup, which can vary from ~20-40 minutes depending on image resolution and topup configuration.

R2.C10: The mention of Figure 4 in the discussion should actually refer to Figure 6.

This has been fixed, and now refers to Figure 7.
