# Supplementary material for: Distortion correction of diffusion weighted MRI without reverse phase-encoding scans or field-maps
Source: PLoS One. 2020 Jul 31;15(7):e0236418. doi: 10.1371/journal.pone.0236418 (PMC7394453; doi:10.1371/journal.pone.0236418)
Supplement: Attachment — Submitted filename: R2_Response2Reviewers.docx [file pone.0236418.s002.docx]

­­Dear Dr. Yap,

Please find attached a revision in response to the concerns raised by the reviewers. Major changes include a thorough grammatical review by native English speaking co-authors of the manuscript as well as by a consultant at Vanderbilt Writing Studio. We have also made minor modifications to text and figure descriptions. The text that directly responds to the reviewers’ concerns is highlighted and annotated below, and is marked in the revised manuscript.

Kind regards,

Kurt Schilling

R1.C1: Check if the statement is correct: "To account for this, the median value of the masked undistorted b0 was scaled such that it matched the masked median value of the undistorted b0". Distorted should be matched to undistorted B0 image?

Yes, this is correct. We now explain why the signal intensity values may be offset, as well as why we chose to scale in this way. This is very similar to what topup does with their “-scale” option, and we only need to get close to the correct intensity for image synthesis (in a similar way topup needs close to similar intensities for susceptibility distortion):

because the intensities for the distorted b0 and undistorted b0 were not guaranteed to match due to the fact that the undistorted b0 was a separate acquisition with a potentially different gain factor. To account for this, the median value of the masked undistorted b0 was scaled such that it matched the masked median value of the undistorted b0 (in a process similar to that done by topup with the *-scale* option).

R1.C2: In Fig. 7, what do different colored markers represent?

The colors represent which dataset that data point came from. We now state this in the figure caption directly:

Each contains 15 datapoints, from 5 HCP subjects (blue), 5 Vanderbilt subjects (black), and 5 BLSA subjects (red).

R1.C3: In Fig. 4, no input is mentioned at the beginning.

We now state this clearly in the text and in the figure caption:

Each U-net has 2 input channels (a T1 image and distorted b0) and a single output channel (synthesized undistorted b0).

R1.C4: Language of the paper needs significant improvement. There are many grammatical errors, such as: "Figure 8 shows that these can correct distortions on datasets that may differ from those the networks were trained on." 'These' should be changed to 'this'.
       "The inverse transforms were used to convert the generated undistorted b0 back into subject space." Geometric transforms do not "convert" images; transforms deform/warp images.

Thank you for the feedback. We want to be sure this work is readable and intuitive. We have made the two recommended changes, and also performed a grammatical review of the manuscript.

The authors have addressed all my concerns.

I just have two minor comments:

R2.C1: In the intro of section 2 the authors state: "in order to provide topup the information necessary to perfectly correct the distorted diffusion data." The word "perfectly" is a bit of an overstatement (no tool is perfect), so I would suggest to remove it.

Thank you for the comments. We agree that the word “perfectly” is probably never appropriate when describing tools. We have removed this from the text.

R2.C2: In the right panel in Figure 5 the dots are grey, not green as stated in the caption of Figure 5.

Green has been changed to gray.
